# Does the Use of Celluloid Infringe the Cumming's Patent ?

**Published:** 1876-01

**Authors:** 


### EDITORIAL, ETC.

---

*Does the use of Celluloid in making Dental Plates infringe the "Cummings Patent."*—DECISION BY JUDGE BLATCHFORD, ON THE QUESTION.—The Dental profession are familiar with the threats that have been made by Josiah Bacon and the Goodyear Dental Vulcanite Co., as to the infringement of the "*Cummings Patent*" by the use of Celluloid, and have been put to much inconvenience thereby, but until lately, we have had no opportunity to answer their complaint.

We have at all times desired a trial upon the merits of the issue between the Goodyear Dental Vulcanite Co. and ourselves, and we have done all we could to have such a trial reached, and to have the issue disposed of, confident that it would be settled in our favor.

Finally in July, 1875, the Company and Mr. Bacon filed their Bill of Complaint in the Circuit Court of the United States for the Southern District of New York, against Dr. Eben M. Flagg, a dentist of New York City, for the purpose of enjoining Dr. Flagg from using Celluloid in the making of Dental Plates.

The Plaintiff's motion was made before Judge Blatchford for a preliminary injunction against Dr. Flagg, and was fully ar, gued by Messrs. E. N. Dickerson and B. F. Lee for the plaintiffs- and by Judge Wm. D. Shipman, Clarence A. Seward and E. Luther Hamilton for Dr. Flagg—every thing that could aid the plaintiffs, either of fact or in other previous decisions in their favor was brought up by the plaintiffs, and after considering the facts and arguments, Judge Blatchford, on December 7th, 1875, denied the motion, and rendered the following opinion:

U. S. CIRCUIT COURT, SOUTHERN DISTRICT OF NEW YORK.

THE GOODYEAR DENTAL VULCANITE CO., AND OTHERS, *vs.* EBEN M. FLAGG.

"I do not find that any decision has been made in regard to the plaintiff's patent which gives to it such a construction as necessarily includes the process and substance used by the defendant. In the Gardiner case the defendant did not compound India-rubber with sulphur, but he compounded India-rubber with Iodine, and he employed heat to harden the rubber. (*Goodyear Dental Vulcanite Co.* vs. *Gardiner*, 4 *Fishers Patent Cases*, 224, 231.)

In the Smith case the view of the Court was that the material to be used under the plaintiff's patent in carrying out the invention patented was to be India-rubber, "and the compounds commonly employed therewith reduced to a soft plastic condition, capable of vulcanization and subsequently vulcanized." (*Goodyear Dental Co.* vs. *Smith*, 5 *Official Gazette of Patent Office* 585.)

It appears, from the description of the process used by the defendant in this suit, that he does not use India-rubber or any substance capable of vulcanization: That the substance he uses is one which is rendered plastic by heat, and is not hardened by heat; That heat is used in the process to soften the substance and render it plastic and not to harden it, and that the substance after being moulded is hardened by being cooled. It is not sufficiently clear that this process is embraced in the claim of the plaintiff's patent to warrant the granting of an injunction until one is awarded as the result of a decree for the plaintiffs on final hearing."

The Celluloid Manufacturing Co. of 295 Ferry Street, Newark. N. J., makes the following request:—We take this opportunity to again request of the members of the Dental profession that they will, in event of any suits being brought against them for the use of Celluloid, send us, without delay, the papers in the case that we may defend the suit, which we will do at our cost.

It is well known that our factory was destroyed by fire in September last, but we are happy to state, that we are again at work, and will be ready to supply dental plates through our agent, S. S. White, Philadelphia, about January, 15th, 1876.

